# Analytical modeling and numerical analysis of thermoelastic damping in ultrathin elastic films due to surface effects

**DOI:** 10.1038/s41598-023-46826-1

**Published:** 2023-11-11

**Authors:** Dianwu Huang, Houren Xiong, Guangying Yang

**Affiliations:** College of Civil Engineering and Architecture, Jiaxing University, Jiaxing, 314001 Zhejiang China

**Keywords:** Engineering, Materials science

## Abstract

Analytical techniques used for estimating thermoelastic damping by incorporating both mechanical and thermal interactions between surfaces and the rest of the bulk are intricate and challenging due to the limited understanding of the damping mechanisms in extra-thin films subjected to forced vibrations. This paper proposes a modified model to analytically calculate the thermoelastic damping of ultrathin elastic films due to surface effects and analyzes the thermoelastic damping variation with different factors through numerical experiments on two materials. The model considers surface stresses derived from the elastic surface theory using Kirchhoff's kinetic hypothesis and determines thermoelastic damping by considering thermal dissipation and elastic potential energy. The results show that surface effects significantly influence the thermoelastic damping of the film, and the specific behavior of a thin film’s thermoelastic damping with respect to film thickness is impacted by various factors, including material property, the variation range of film thickness, and the forced vibration frequency. This study provides insights into the thermoelastic damping behavior of thin films and has important implications for the development of nanoscale oscillators in MEMS or NEMS systems.

## Introduction

Microscale and nanoscale plates or shells serve as essential components in microelectromechanical systems (MEMS) and nanoelectromechanical systems (NEMS)^1,2^. Owing to their diminutive dimensions, these structures exhibit unique mechanical and thermal characteristics that deviate from their macroscopic counterparts. Comprehensive insights into the physical and mechanical behaviors of these microstructures and nanostructures are therefore indispensable and have garnered significant attention in the research community^3–7^.

The surfaces, as well as regions adjacent to the surfaces of solid films, are characterized by a minuscule thickness where atomic arrangements differ from the bulk material. Consequently, surface effects play a pivotal role in modulating the mechanical properties of the film, especially when the surface-to-volume ratio becomes increasingly significant^8–13^. Both experimental investigations and atomic-level simulations have confirmed that the surface structure and properties, such as surface stiffness, differ substantially from those in the bulk material^14–16^. Gurtin and Murdoch rigorously developed a general model that accounts for surface elasticity by conceptualizing the surfaces as two-dimensional membranes with distinct properties from the bulk material, adhering to the latter without any slippage^17–19^. Building upon the Gurtin–Murdoch surface elasticity model, Huang et al.^20^ formulated an analytical framework that incorporates the effects of curvature and classical inertia to study the nonlinear vibrations of simply supported thin films. The frequency of the film, rendered dimensionless for comparison, was computed and revealed significant deviations due to surface effects. Moreover, subsequent research has established that the mechanical and nonlinear vibrational properties of nanoscale thin films are intrinsically tied to their geometric features, particularly the size of the film, in numerous MEMS and NEMS applications^20–25^.

One noteworthy phenomenon is the generation of strain-induced heat in film-like oscillators undergoing high-frequency vibrations due to resonant forces, a manifestation of the well-understood thermoelastic effect. This results in a thermal gradient across the film, leading to a decrease in temperature in tensioned regions and an increase in temperature in compressed regions. Such heat transfer within the structure can induce thermoelastic damping, which in turn can attenuate the vibrational motion of the oscillator^26^. Recognizing thermoelastic damping as a critical attribute, engineers and researchers have emphasized its role in the structural design of various electromechanical and nanomechanical systems^27–30^. However, a comprehensive analytical approach that integrates both mechanical and thermal interactions between surface and bulk regions for thermoelastic damping prediction is lacking. Existing studies on damping mechanisms primarily rely on empirical or semi-empirical methodologies. Furthermore, the influence of variables such as film size, material properties^31^, and forced vibration frequency on thermoelastic damping, especially in the context of surface effects, remains inadequately understood.

This study aims to formulate an analytical model to quantify thermoelastic damping in strip films, taking into account the impact of surface effects. The model employs surface stress equations based on elastic surface theory, using Kirchhoff's kinetic hypothesis as the foundational principle. Thermoelastic damping is then evaluated through the calculation of thermal dissipation and peak elastic potential energy. The insights gained from understanding thermoelastic damping will contribute to the informed and optimized design of thin film components in MEMS and NEMS systems.

## Problem formulation

This study employed Kirchhoff's modified plate theory^32^ to formulate the heat conduction equation for thin-film structures, incorporating displacement based on the principle of minimizing heat flow potential. Kirchhoff's modified plate theory serves as a mathematical model that elucidates the behavior of thin plates under mechanical loads. Concurrently, a thermoelastic damping model was developed, accounting for surface effects. This model is predicated on the ratio of energy dissipation to the maximum elastic energy within one oscillation cycle. Subsequent to establishing this thermoelastic damping model, a parameter analysis was conducted to deeply understand the impact of surface effects on thermoelastic damping.

### Governing equations for the transverse linear steady-state vibration of elastic film

**Figure 1:**
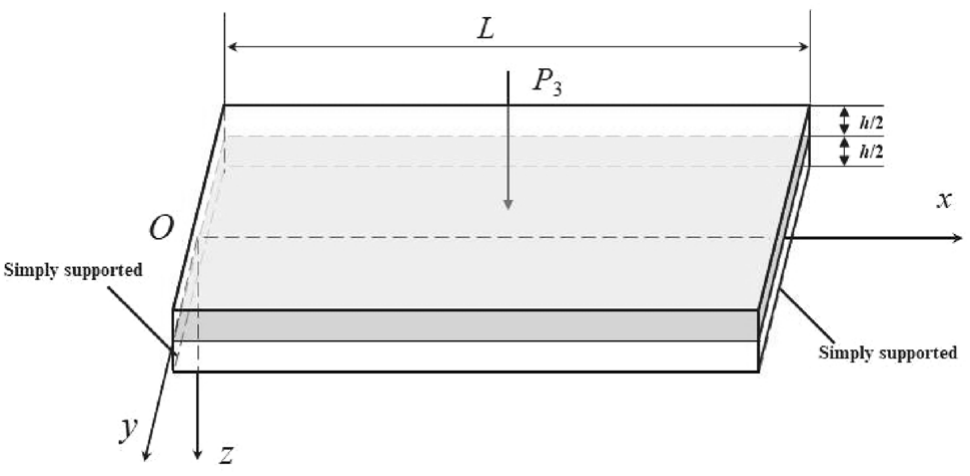
A schematic of the periodic vibration of a simply supported film or nanoplate.

Consider a rectangular strip film with a constant thickness *h* and dimensions *L* × $\infty$ along the x-axis and y-axis. This film is subject to a harmonic load given by ${\varvec{P}}_{3} = {\varvec{P}}_{0} {\varvec{e}}^{{\user2{i\omega t}}}$ (see Fig. 1). The edges along the y-axis are simply supported. Here, *u*_*x*_, *u*_*y*_, and ${\varvec{w}}$ represent the displacements along the *x*-, *y*-, and *z*-directions, respectively. Given the film’s infinite length along the y-axis, the displacement in this direction is zero ($u_{y} = 0$).

The boundary conditions along these edges are expressed in:

1 $$ u_{x} = w_{,xx} = 0\quad at\quad x = 0,L $$

Adopting Kirchhoff's assumptions, the equilibrium equations for membrane force and bending moments can be expressed as:

2 $$  \begin{aligned} & N_{\alpha \beta ;\beta }^{*} = 0 \\ & M_{\alpha \beta ;\alpha \beta }^{*} + u_{3,\alpha \beta } N_{\alpha \beta } + P_{3} - I\ddot{u}_{3} = 0 \\ \end{aligned} $$

The usual summation convention for repeated indices will be adopted with Greek indices α or β taking the values of 1 and 2. A comma represents differentiation with respect to the suffix index, where $I = \mathop \int \limits_{ - h/2}^{h/2} \rho dx_{3} = \rho h$.

The precise expressions of the membrane force and the bending moment with the surface effects are given by Huang et al.^33,34^:

2 $$ \begin{aligned} & N_{\alpha \beta }^{*} = 2\tau_{0} \left( {\delta_{\alpha \beta } + u_{\alpha ,\beta }^{0} } \right) + D_{1} \left[ {\left( {1 - \nu } \right)\left( {1 + \frac{{l_{1} }}{h}} \right)\varepsilon_{\alpha \beta }^{0} + \nu \left( {1 + \frac{{l_{2} }}{h}} \right)\varepsilon_{\gamma \gamma }^{0} \delta_{\alpha \beta } } \right], \\ & M_{\alpha \beta }^{*} = - D_{2} \left[ {\left( {1 - \nu } \right)\left( {1 + 3\frac{{l_{1} }}{h}} \right)u_{3,\alpha \beta } + \nu \left( {1 + 3\frac{{l_{2} }}{h}} \right)u_{3,\gamma \gamma } \delta_{\alpha \beta } } \right] \\ \end{aligned} $$

where $l_{1} = 4\frac{{\left( {1 + \nu } \right)\left( {\mu_{0} - \tau_{0} } \right)}}{E}$ and $l_{2} = 2\frac{{\left( {1 - \nu^{2} } \right)\left( {\lambda_{0} + \tau_{0} } \right)}}{E\nu }$ are the intrinsic length scales; $\varepsilon_{\alpha \beta }^{0}$ and $u_{\alpha }^{0}$ indicate the strain and displacement of the midplane respectively. $\mu_{0}$, $\tau_{0}$, and $\lambda_{0}$ represent the surface material constants; $v$ denotes Poisson’s ratio; $E$ is the modulus of elasticity; $\delta_{\alpha \beta }$ is Kronecker delta: $\delta_{\alpha \beta } = 1$ for $\alpha = \beta$ and $\delta_{\alpha \beta } = 0$ for $\alpha \ne \beta$; $D_{1} = \frac{Eh}{{\left( {1 - \nu^{2} } \right)}}$; $D_{2} = \frac{{Eh^{3} }}{{12\left( {1 - \nu^{2} } \right)}}$.

We then introduce the following dimensionless quantities:

3 $$ \overline{w} = \frac{w}{h},\quad \xi = \frac{x}{h},\quad s = \frac{L}{h},\quad P_{0} = \frac{{12P\left( {1 - v^{2} } \right)}}{AE},\quad \overline{\omega } = \frac{1}{{\overline{t}}} $$

where $$\overline{t} = t\sqrt {\frac{E}{{12\rho \left( {1 - v^{2} } \right)s^{4} h^{2} }}}$$ and $$A = 1 + 3\left( {1 - v} \right)\frac{{l_{1} }}{h} + 3v\frac{{l_{2} }}{h}$$.

The boundary conditions in Eq. (1) transform into dimensionless form as:

4 $$ \overline{w} = \overline{w}_{,\xi \xi } = 0\quad at\quad \xi = 0,s $$

Substituting Eq. (3) into Eq. (2) and making it dimensionless yield the dimensionless control formula for the transverse linear steady-state vibration of the elastic film as follows:

5 $$ \overline{w}_{,\xi \xi \xi \xi } + \frac{1}{{As^{4} }}\frac{{\partial^{2} \overline{w}}}{{\partial \overline{t}^{2} }} = P_{0} e^{{i\overline{\omega }\overline{t}}} $$

For a linear steady-state vibration, the elastic film's deflection and the resonant force frequency align without phase difference. Thus, the film's deflection solution is:

6 $$ \overline{w} = \overline{w}_{0} e^{{i\overline{\omega }\overline{t}}} $$

Substituting Eq. (6) into the transverse vibration Eq. (5), the control equation under mode $e^{i\bar{\omega} \bar{t} }$ is obtained as follows:

7 $$ \bar{w}_{0,\xi \xi \xi \xi } - \frac{{\bar{\omega}^{2} }}{{As^{4} }}\bar{w}_{0} = P_{0}$$

By solving Eq. (7), $\bar{w}_{0}$ can be expressed as:

8 $$ \bar{w}_{0} = - \frac{{P_{0} As^{4} }}{{\bar{\omega}^{2} }} + C_{1} \cos \frac{{\sqrt {\bar{\omega} } }}{{A^{\frac{1}{4}} s}}\xi + C_{3} \sin \frac{{\sqrt {\bar{\omega} } }}{{A^{\frac{1}{4}} s}}\xi + C_{2} e^{{ - \frac{{\sqrt {\bar{\omega} } }}{{A^{\frac{1}{4}} s}}\xi }} + C_{4} e^{{\frac{{\sqrt {\bar{\omega} } }}{{A^{\frac{1}{4}} s}}\xi }}$$

Using the boundary conditions in Eq. (4), we can define constants $C_{1}$. , $C_{2}$, $C_{3}$. , and $C_{4}$ in Eq. (8) as:

9 $$  \left\{ {\begin{array}{*{20}l} {C_{1} = \frac{{P_{0} As^{4} }}{{2\bar{\omega}^{2} }}} \\ {C_{2} = \frac{{P_{0} As^{4} e^{{\sqrt {\bar{\omega} } }} }}{{2\left( {1 + e^{{\sqrt {\bar{\omega} } }} } \right)\bar{\omega}^{2} }}} \\ {C_{3} = - \frac{{P_{0} As^{4} \left[ {\cot \left( {\sqrt {\bar{\omega} } } \right) - \csc \left( {\sqrt {\bar{\omega} } } \right)} \right]}}{{2\bar{\omega}^{2} }}} \\ {C_{4} = \frac{{P_{0} As^{4} }}{{2\left( {1 + e^{{\sqrt {\bar{\omega} } }} } \right)\bar{\omega}^{2} }}} \\ \end{array} } \right. $$

### Heat conduction equation

During the linear steady-state vibration of a simply supported film subjected to resonant forces $Pe^{i\omega t}$, the film vibrates at its natural frequency, leading to alternate compression and tension in different parts of the film. This mechanical energy, during these vibrations, is partially transformed into thermal energy due to frictional forces and inherent material damping. Compressed regions generate heat from frictional forces and material deformation, resulting in a temperature rise. While tensioned regions store energy with minimal heat generation, causing a temperature decrease. Consequently, a temperature gradient is established within the film, inducing heat transfer.

In micro or nanomechanical systems, where the elastic film acts as a high-frequency resonator, the vibrations of such thin films can be viewed as an adiabatic process. This is because the high vibration frequencies typically prevent rapid heat exchange with the surrounding environment. Without considering an internal heat source, the heat conduction equation within the film can be derived using the minimum energy principle applied to temperature changes and subsequent integration. It is expressed as:

10 $$ \theta_{,ii} - \frac{1}{\kappa }\dot{\theta } - \frac{{E\alpha_{t} T_{0} }}{{\left( {1 - \upsilon } \right)}}\dot{e} = 0 $$

where $\theta$ indicates the temperature change, $T_{0}$ is the initial temperature, $e = \varepsilon_{ii}$, $\kappa = \frac{k}{{\rho c_{e} }}$ represents the thermal diffusion coefficient, $k$ denotes thermal conductivity, $\rho$ is the material density, and $c_{e}$ stands for the specific heat capacity.

Given the absence of an internal heat source in the film, heat generation can solely arise from the work exerted by external forces. By adhering to Kirchhoff’s assumption, the positive strain effect in the thickness direction is disregarded, leading to the condition: $\varepsilon_{zz} = 0$. In the context of thin film vibration incited by external resonant forces, strain-induced heat arising from these forces is the primary cause of the temperature gradient. When addressing heat transfer within the film, it can be assumed that the temperature fluctuation along the film’s thickness greatly surpasses any in-plane changes. Hence, in the heat conduction equation, the mid-plane's temperature deviation can be disregarded to streamline computations. Furthermore, when analyzing the small-amplitude nonlinear vibration characteristics of thin films, the film's displacements within the x–y plane are minimal in contrast to the amplitude variations in the z-direction. This is primarily due to the simply supported boundary conditions along the film's edges. Therefore, in-plane displacements are insignificant and can be overlooked.

11 $$ e = - z\left( {w_{,xx} + w_{,yy} } \right) + \frac{1}{2}\left( {w_{,x}^{2} + w_{,y}^{2} } \right) $$

Since the elastic film, as an oscillator in the microelectromechanical system, vibrates at a high frequency and exchanges heat with the surrounding environment very slowly, the thermal boundary condition of the film can be approximated to an adiabatic process. Using the dimensionless quantity expression in Eq. (3) and introducing the dimensionless variable $\overline{\theta } = \alpha_{t} \theta$, the dimensionless thermal boundary condition can be defined as:

12 $$  \frac{\partial \bar{\theta} }{{\partial \bar{z} }} = 0\quad at\quad \bar{z} = \pm \frac{1}{2} $$

The linear dimensionless heat conduction equation is also given by:

13 $$  \frac{{\partial^{2} \bar{\theta} }}{{\partial \bar{z}^{2} }} - \frac{1}{\kappa }\sqrt {\frac{E}{{12\rho \left( {1 - v^{2} } \right)s^{4} }}} \frac{\partial \bar{\theta} }{{\partial \bar{t} }} - \frac{{E\alpha_{t}^{2} T_{0} }}{1 - v}\frac{\partial }{\partial \bar{t} }\left( { - \bar{w}_{,\xi \xi } \bar{z} } \right) = 0 $$

### Temperature change distribution

When the elastic film is under a linear steady-state vibration, the frequency of the vibration is constant and equal to the frequency of the applied resonant force. As a result, the strain within the film also oscillates at the same frequency. This strain causes a change in temperature, known as the Peltier effect^35^, which occurs at the same frequency as the vibration, which is also equal to the frequency of the applied resonant force. There is only a phase difference between it and the resonant force, so the dimensionless temperature change ($\bar{\theta}$) can be defined as:

When the elastic film undergoes linear steady-state vibration, the vibration frequency remains constant, matching the frequency of the externally applied resonant force. Consequently, the induced strain within the film oscillates harmoniously at this consistent frequency. This strain-triggered temperature variation, attributed to the Peltier effect^35^, oscillates in sync with the vibration frequency, which, in turn, aligns with the frequency of the applied resonant force. Given that the only deviation between them is a phase shift, the dimensionless temperature change ($\bar{\theta}$), can be articulated as

14 $$  \bar{\theta} = \bar{\theta}_{0} e^{i\bar{\omega} \bar{t} } $$

Substituting Eq. (14), the amplitude expression, i.e., Eq. (6), and the solution to $\bar{w}_{0}$, i.e., Eq. (8), into the heat conduction equation, Eq. (13), yields:

15 $$  \frac{{\partial^{2} \bar{\theta}_{0} }}{{\partial \bar{z}^{2} }} - \gamma_{1} \bar{\theta}_{0} + \gamma_{2} \bar{z} = 0 $$

where $$ \gamma_{1} = \frac{{i\bar{\omega} \left[ {\frac{E}{{12\rho \left( {1 - v^{2} } \right)}}} \right]^{1/2} }}{{\kappa s^{2} }}$$ and $$ \gamma_{2} = \frac{{i\bar{\omega} C_{5} E\alpha_{t}^{2} T_{0} }}{{\left( {1 - v} \right)}}$$.

$C_{5}$ is also expressed by:

16 $$  C_{5} = \bar{w}_{0,\xi \xi } = - \frac{{P_{0} As^{4} }}{{\bar{\omega}^{2} }} + \frac{\bar{\omega} }{{A^{\frac{1}{2}} s^{2} }}\left( { - C_{1} \cos \frac{{\sqrt {\bar{\omega} } }}{{A^{\frac{1}{4}} s}}\xi - C_{3} \sin \frac{{\sqrt {\bar{\omega} } }}{As}\xi + C_{2} e^{{ - \frac{{\sqrt {\bar{\omega} } }}{{A^{\frac{1}{4}} s}}\xi }} + C_{4} e^{{\frac{{\sqrt {\bar{\omega} } }}{{A^{\frac{1}{4}} s}}\xi }} } \right) $$

where $C_{1}$, $C_{2}$, $C_{3}$, and $C_{4}$ are determined by Eq. (9).

Solving Eq. (15) gives the temperature change distribution ($\bar{\theta}_{0}$) as follows:

17 $$  \bar{\theta}_{0} = \frac{{\gamma_{2} }}{{\gamma_{1} }}\bar{z} + c_{1} e^{{\sqrt {\gamma_{1} } \bar{z} }} + c_{2} e^{{ - \sqrt {\gamma_{1} } \bar{z} }} $$

Using the thermal boundary conditions in Eq. (12), we can determine the constant in Eq. (17) by:

18 $$ \left\{ {\begin{array}{*{20}c} {c_{1} = - \gamma_{2} \sqrt {\gamma_{1}^{ - 3} } {\text{sech}}\left( {\frac{{\sqrt {\gamma_{1} } }}{2}} \right)} \\ {c_{2} = \gamma_{2} \sqrt {\gamma_{1}^{ - 3} } {\text{sech}}\left( {\frac{{\sqrt {\gamma_{1} } }}{2}} \right)} \\ \end{array} } \right. $$

### Thermoelastic damping model

For issues involving periodic vibration damping effects, the system's thermoelastic damping, denoted as ($\psi$), is formulated based on the proportion of the total thermal dissipation, Δ $W$, to the peak elastic potential energy, *W*, over a single cycle. As proposed by Bishop and Kinra et al.^36^, the relationship can be expressed as:

19 $$ \psi = \frac{\Delta W}{W} $$

The maximum elastic potential energy $$W $$ of an elastic film influenced by surface effects and subjected to resonant forces over one cycle can be described by the relation $$W = \frac{1}{2}\mathop \int \limits_{V} \sigma_{ij} \varepsilon_{ij} dV$$. This energy encompasses the maximum elastic potential energies of the film's body $$W^{V}$$, its upper surface $$W^{{S^{ + } }}$$, and its lower surface $$ W^{{S^{ - } }}$$ within that cycle. Likewise, the variation in the elastic potential energy $$\Delta W$$ over one cycle is the summation of the changes in the elastic potential energies of the film's body Δ*W*^*V*^, its upper surface Δ*WS*^+^, and its lower surface $$\Delta W^{{S^{ - } }}$$. Consequently, the thermoelastic damping, quantified in terms of energy for a single cycle of an elastic film subjected to resonant forces with the presence of surface effects, is expressed by:

20 $$ \psi = \frac{{\Delta W^{V} + \Delta W^{{S^{ + } }} + \Delta W^{{S^{ - } }} }}{{W^{{S^{ + } }} + W^{{S^{ - } }} + W^{V} }} $$

According to the results of Bishop and Kinra et al.^36^, Eq. (21) can express the heat dissipation on the upper surface in one cycle by taking the upper surface stress as $\tau_{\xi }^{ + }$:

21 $$  \Delta W^{{S^{ + } }} = - \pi \mathop \int \limits_{0}^{s} \bar{\tau}_{\xi }^{ + } Im\left( {\bar{\theta}^{ + } } \right)d\xi $$

The heat dissipation of the lower surface in one cycle is also given by:

22 $$  \Delta W^{{S^{ - } }} = - \pi \mathop \int \limits_{0}^{s} \bar{\tau}_{\xi }^{ - } Im\left( {\bar{\theta}^{ - } } \right)d\xi $$

where

23 $$  \bar{\tau}_{\xi \xi }^{ \pm } = \frac{{\tau_{0} + 2\left( {\mu_{0} - \tau_{0} + \lambda_{0} } \right)}}{Eh}\left( { \pm \frac{1}{2}C_{5} } \right) $$

24 $$  \bar{\theta}^{ \pm } = - \frac{{\gamma_{2} }}{{\gamma_{1} }}\left( { \pm \frac{1}{2}} \right) + c_{1} e^{{\sqrt {\gamma_{1} } \left( { \pm \frac{1}{2}} \right)}} + c_{2} e^{{ - \sqrt {\gamma_{1} } \left( { \pm \frac{1}{2}} \right)}} $$

The maximum elastic potential energy of the upper surface is defined as:

25 $$  W^{{S^{ + } }} = - \frac{1}{4}\mathop \int \limits_{0}^{s} \bar{\tau}_{\xi \xi }^{ + } C_{5} d\xi $$

The maximum elastic potential energy of the lower surface is expressed by:

26 $$  W^{S - } = \frac{1}{4}\mathop \int \limits_{0}^{s} \bar{\tau}_{\xi \xi }^{ - } C_{5} d\xi $$

Introducing the body stress, $\bar{\sigma}_{\xi } = - \frac{{\bar{w}_{0,\xi \xi } \bar{z} }}{{\left( {1 - v^{2} } \right)}}$, we can obtain the internal heat dissipation in one cycle from:

27 $$  \Delta W^{V} = - \pi \mathop \int \limits_{{ - \frac{1}{2}}}^{\frac{1}{2}} \mathop \int \limits_{0}^{s} \bar{\sigma}_{\xi } Im\left( {\bar{\theta} } \right)d\xi d\bar{z} $$

The maximum elastic potential energy in one cycle is also defined as:

28 $$  W^{V} = \frac{1}{{2\left( {1 - v^{2} } \right)}}\mathop \int \limits_{{ - \frac{1}{2}}}^{\frac{1}{2}} \mathop \int \limits_{0}^{s} \left( { - \bar{z} C_{5} } \right)^{2} d\xi d\bar{z} $$

Substituting the energy dissipation expressions for one cycle, i.e., Eqs. (21), (22), and (27), and the maximum elastic energy expressions for one cycle, i.e., Eqs. (25), (26), and (28), into the thermoelastic damping expression, Eq. (20), defines the thermoelastic damping as:

29 $$  \psi = \frac{{ - \pi \left[ {\frac{1}{{24\left( {1 - v^{2} } \right)}}\mathop \int \nolimits_{{ - \frac{1}{2}}}^{\frac{1}{2}} \mathop \int \nolimits_{0}^{s} \left( { - \bar{z} C_{5} } \right)Im\left( {\bar{\theta} } \right)d\xi d\bar{z} + \mathop \int \nolimits_{0}^{s} \bar{\tau}_{\xi }^{ + } Im\left( {\bar{\theta}^{ + } } \right) + \bar{\tau} \bar{\xi} Im\left( {\bar{\theta}^{ - } } \right)d\xi } \right]}}{{\left( {\frac{1}{{24\left( {1 - v^{2} } \right)}} + \frac{1}{4}\frac{l}{h}} \right)\mathop \int \nolimits_{0}^{s} C_{5}^{2} d\xi }} $$

where intrinsic length scale $l = \left( {2\mu_{0} - \tau_{0} + \lambda_{0} } \right)/E$, when the surface effect is neglected, then $\tau_{0} \to 0$, $\lambda_{0} \to 0$ and $\mu_{0} \to 0$, the equations take the same as those without surface effect. To calculate the thermoelastic damping, the key parameters in Eq. (30) mainly include Poisson’s ratio $v$, film width-to-thickness ratio $s$, thickness *h*, intrinsic length scale *l*, and C_5_.

## Numerical experiments

To clarify the thermoelastic damping characteristics exhibited by elastic films affected by surface effects, we undertake numerical experiments. These cover two distinct cases involving the linear steady-state vibration of elastic films under the action of resonant forces. A thorough parameter analysis was carried out to understand the influence of essential parameters on thermoelastic damping. These parameters include attributes such as film thickness, the ratio of film width to thickness, material constants, and the frequency of the forced vibration. Additionally, for a comparative viewpoint, our findings from each case were contrasted with the results from the linear steady-state vibration of elastic films subjected to resonant forces without considering any surface effects. Additionally, to provide a comparative perspective, our findings from each scenario are juxtaposed against the outcomes derived from the linear steady-state vibration of elastic films subjected to resonant forces but in the absence of any surface effects. This comparative analysis aims to highlight the differential impact of surface effects on the film's behavior and to validate the relevance and significance of incorporating such effects in our computational models.

### Material parameters

Two representative materials (Set I and Set II) with distinct properties given by Gurtin and Murdoch^17^ were used for analyzing the size-dependent thermoelastic damping of the strip film considering surface effects under periodic vibration. The details of materials are as follows:

*Set I* a glass substrate coated with 100 nm thick layer of iron (Material I)

30 $$ \begin{aligned} & E = 5.625 \times 10^{10} \frac{{\text{N}}}{{{\text{m}}^{2} }},\quad \nu = 0.25,\quad \rho = 3 \times 10^{3} \frac{{{\text{kg}}}}{{{\text{m}}^{3} }}, \\ & \lambda_{0} = 7 \times 10^{3} \frac{{\text{N}}}{{\text{m}}},\quad \mu_{0} = 8 \times 10^{3} \frac{N}{m},\quad \tau_{0} = 110\frac{{\text{N}}}{{\text{m}}}, \\ & \rho_{0} = 7 \times 10^{ - 4} \frac{{{\text{kg}}}}{{{\text{m}}^{2} }},\quad \alpha_{t} = \frac{{5.8 \times 10^{ - 6} }}{ \circ C},\quad \kappa = 7.29 \times 10^{ - 5} \frac{{{\text{m}}^{2} }}{{\text{s}}} \\ \end{aligned} $$

*Set II* a freshly cleaved surface with no additional coating (Material II)

31 $$ \begin{aligned} & E = 17.73 \times 10^{10} \frac{{\text{N}}}{{{\text{m}}^{2} }},\quad \nu = 0.27,\quad \rho = 7 \times 10^{3} \frac{{{\text{kg}}}}{{{\text{m}}^{3} }}, \hfill \\ & \lambda_{0} = - 8\frac{{\text{N}}}{{\text{m}}},\quad \mu_{0} = 2.5\frac{{\text{N}}}{{\text{m}}},\quad \tau_{0} = 1.7\frac{{\text{N}}}{{\text{m}}}, \hfill \\ & \rho_{0} = 7 \times 10^{ - 6} \frac{{{\text{kg}}}}{{{\text{m}}^{2} }},\alpha_{t} = \frac{{5.8 \times 10^{ - 6} }}{{^{{\circ }} C}},\quad \kappa = 7.29 \times 10^{ - 5} \frac{{{\text{m}}^{2} }}{{\text{s}}} \end{aligned} $$

The two materials exhibit different surface material constants, with the second material having a significantly smaller and negative intrinsic length scale *l* (e.g., $- 2.651 \times 10^{ - 11}$) than the first ($4.069 \times 10^{ - 7}$).

### Thermoelastic damping with surface effect

**Figure 2:**
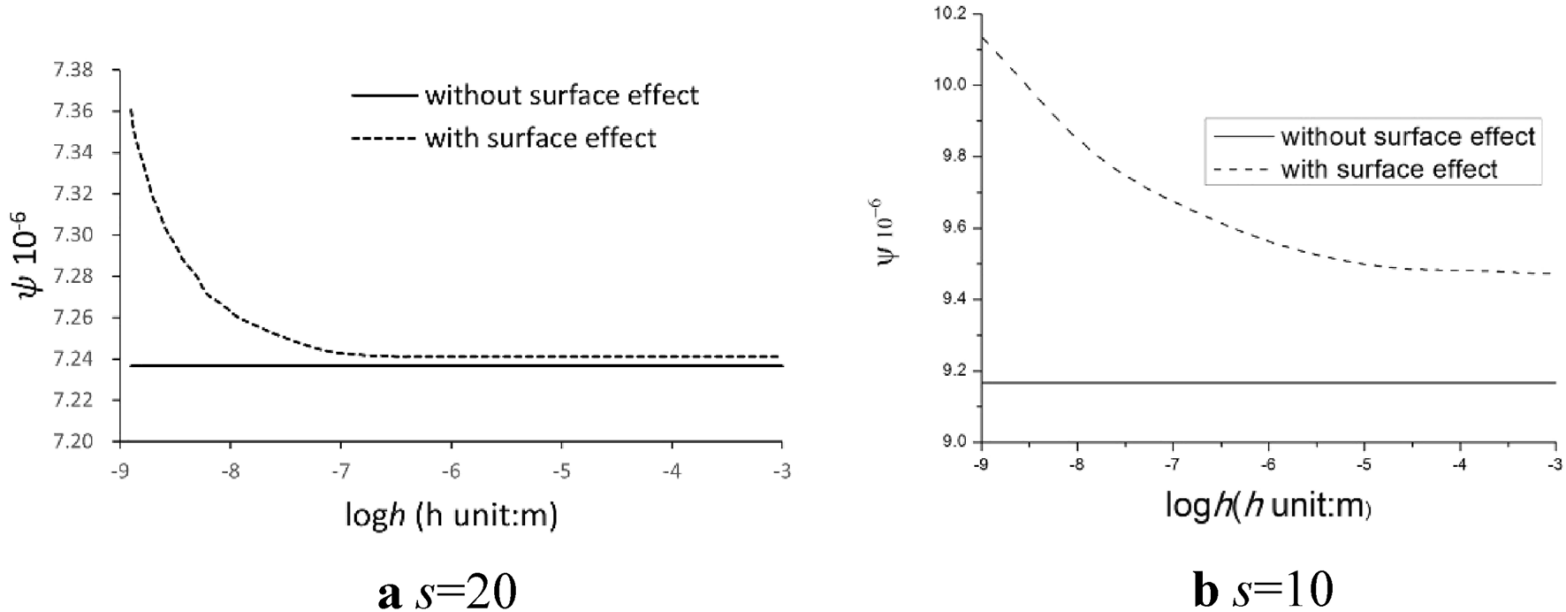
The relationship between the thermoelastic damping and thickness of the thin film for Material I at different fixed width-to-thickness ratios (**a**) *s* = 20 and (**b**) *s* = 10 under the same dimensionless frequency $\overline{\omega } = 10^{4}$.

**Figure 3:**
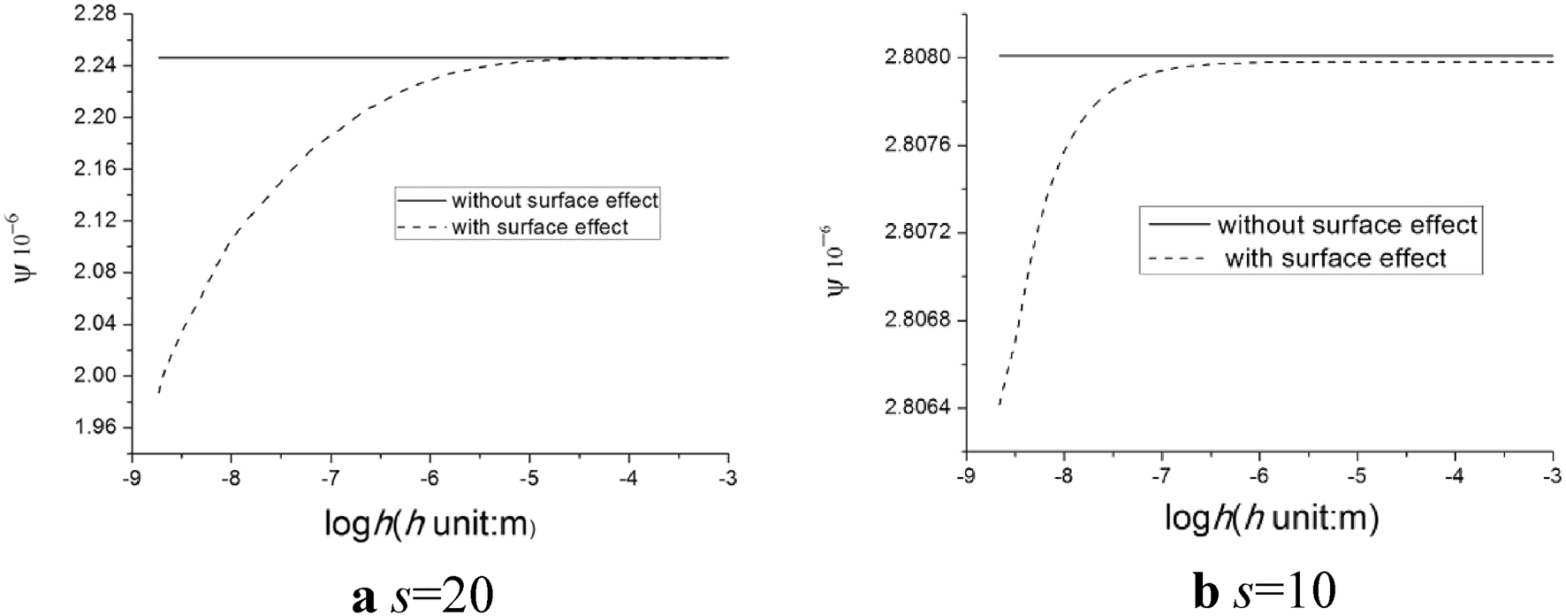
The relationship between the thermoelastic damping and thickness of the thin film for Material II with different fixed width-to-thickness ratios (**a**) *s* = 20 and (**b**) *s* = 10 under the same dimensionless frequency $\overline{\omega } = 10^{4}$.

Results using Eq. (30) indicate that for both materials, notable surface effects emerge at varying thickness values. Figures 2 and 3 illustrate characteristic outcomes for Material I and Material II with width-to-thickness ratios *s* = 20 and *s* = 10 at a dimensionless frequency of 10^4^. It was observed that the thermoelastic damping of a film, when accounting for surface effects, fluctuates with its thickness. Conversely, when excluding surface effects, the thermoelastic damping remains unaffected by the thickness. This observation demonstrating a strong relationship between film thickness and thermoelastic damping under the influence of surface effects, regardless of the vibration frequency. For both Material I and II, when surface effects are disregarded, the constant thermoelastic damping values are 9.17 × 10^−6^ m and 2.81 × 10^−6^ m respectively. When considering surface effects for Material I, the peak thermoelastic damping reaches 10.14 × 10^−6^ m. This value decreases as thickness grows, ultimately stabilizing at 9.47 × 10^−6^ m at *s* = 10. In contrast, for Material II, the thermoelastic damping exhibits a slight growth with an increase in thickness, rising from 2.806 × 10^−6^ m to 2.808 × 10^−6^ m at *s* = 10. The maximum value for thermoelastic damping is remarkably similar to the value achieved without surface effect.

As a material’s size reduces, surface effects become increasingly pronounced, especially as the ratio of surface area to bulk volume rises. At nanoscales, a material’s surface area can significantly dictate its bulk properties. Surface material constants define a material's surface attributes, which may differ from its inherent bulk properties. Addressing these surface material constants becomes vital when examining thin films or nanostructures due to their potential significant impact on the material's attributes and performance. There exists a notable difference between the material constants of Material I and Material II. Figures 2 and 3 further highlight that Material I exhibit a considerably greater thermoelastic damping than Material II, regardless of the presence or absence of surface effects. To illustrate, without considering surface effects, the thermoelastic damping of Material I is 9.17 × 10^−6^ m, which is approximately 3.3 times greater than that of Material II when *s* = 10.

### Impact of width-to-thickness ratio and film thickness

The significance of the surface effect in strip films become evident when the thickness of these films reduces to the nanometer scale. To explore the influence of thickness on the surface effect, we maintained a constant width-to-thickness ratio *s*, for both Material I and Material II. Two specific width-to-thickness ratios were assessed, namely *s* = 10 and *s* = 20, at dimensionless resonant force frequencies of 10^5^ and 10^4^. We present the relationship between thermoelastic damping and film thickness (for both Material I and Material II) at the resonant force 10^4^ as both dimensionless frequencies reveal similar data trends.

Figure 2 illustrates that when the surface effect is excluded, the consistent thermoelastic damping value for a thin film with *s* = 10 is 9.168 × 10^−6^ m. This is 27% greater than that of the film with *s* = 20. This observation suggests that a film with a smaller width (relative to its thickness) tends to exhibit a higher thermoelastic damping when the thickness remains unchanged. Incorporating the surface effects into the analysis, specifically through Eq. (30), and accounting for surface material constants of Material I (i.e., $\tau_{0}$, $\lambda_{0}$ and $\mu_{0}$), reveals that the narrower film still displays higher thermoelastic damping compared to its wider counterpart. Moreover, thermoelastic damping for both films decrease exponentially with increasing thickness. As an instance, for the Material I film with s = 20, thermoelastic damping values reduce by 1.63%, from 7.361 × 10^−6^ m to 7.241 × 10^−6^ m as thickness escalates from 10^−9^ m to 10^−6^ m. Meanwhile, for the narrower film with s = 10, thermoelastic damping values decrease by 6.54%, from 10.136 × 10^−6^ m to 9.473 × 10^−6^ m. Similar findings are also noted for Material II, as depicted in Fig. 3. Specifically, thermoelastic damping values rise by 13.04% and 0.06% for *s* = 20 and *s* = 10.

### Thermoelastic damping with the impact of dimensionless frequency

**Figure 4:**
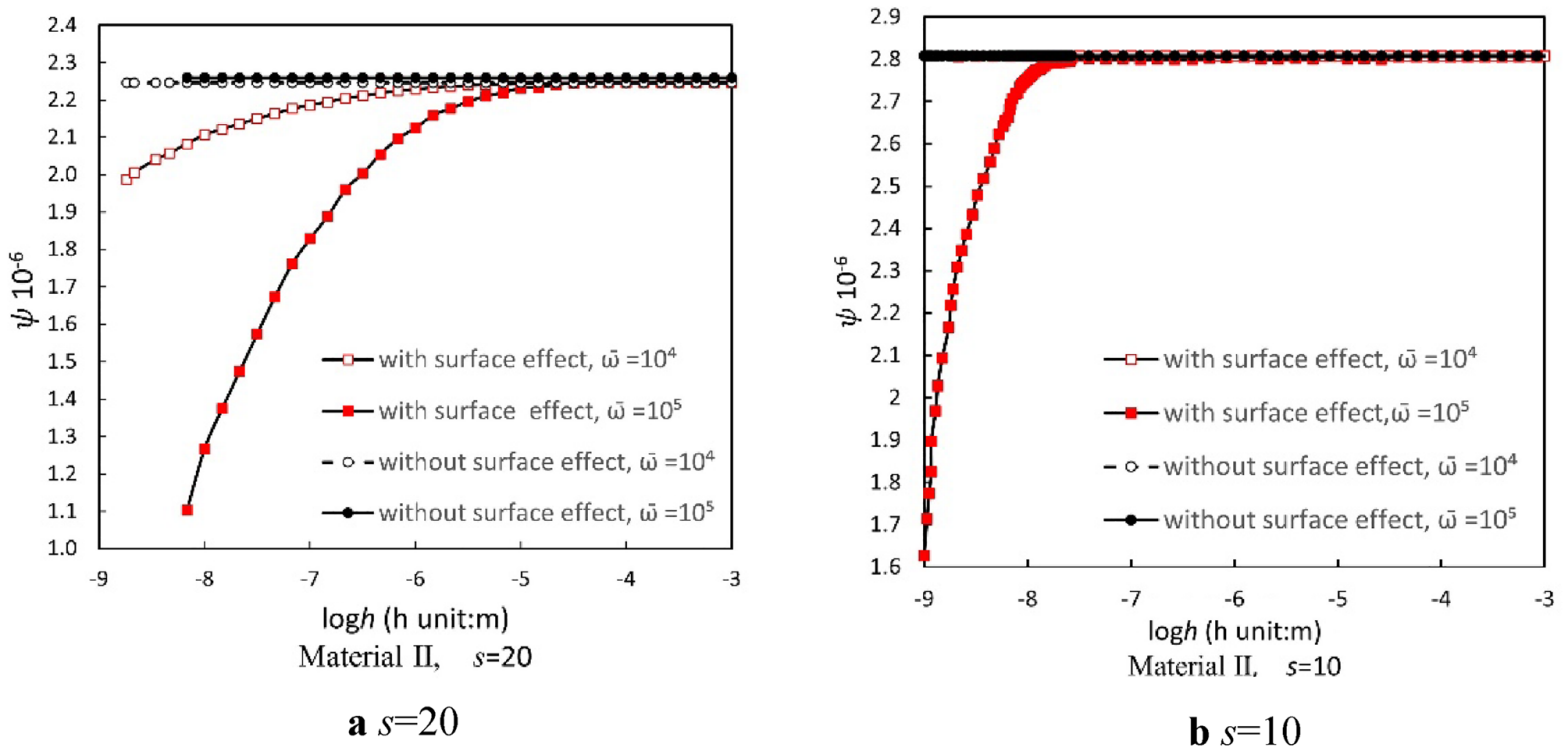
The relationship between the thermoelastic damping and thickness of Material II with a fixed width-to-thickness ratio (**a**) *s* = 20 and (**b**) *s* = 10 under the two dimensionless frequencies $\overline{\omega } = 10^{4}$ and $\overline{\omega } = 10^{5}$.

**Figure 5:**
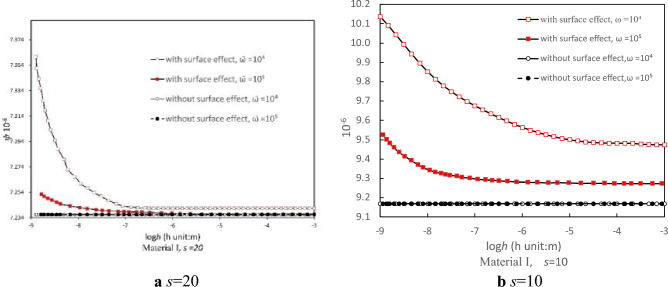
The relationship between the thermoelastic damping and thickness of Material I with a fixed width-to-thickness ratio (**a**) *s* = 20 and (**b**) *s* = 10 under the two dimensionless frequencies $\overline{\omega } = 10^{4}$ and $\overline{\omega } = 10^{5}$.

Figure 4 illustrates the behavior of Material II under the influence of surface effects for width-to-thickness ratios *s* = 20 and *s* = 10. Notably, as the film thickness increases, thermoelastic damping also rises regardless of the vibration frequency. However, the vibration frequency plays a significant role in this increase. Specifically, a higher vibration frequency leads to a more pronounced change in thermoelastic damping due to the surface effect, especially in the thickness range of 10^−9^ to 10^−3^ mm. In contrast, the behavior of Material I, as depicted in Fig. 5, is different. For width-to-thickness ratios *s* = 20 or *s* = 10, the variations in thermoelastic damping, when influenced by surface effects, are less pronounced at higher frequencies.

## Discussion

This study introduces a modified continuum model for an elastic film with nanoscale thickness, incorporating the surface effect, and explores its thermoelastic damping variation influenced by different parameters. By employing the principle of heat flow potential minimization for the periodic vibration of simply supported microscale/nanoscale films, we derive an explicit exact solution for thermoelastic damping. Our analytical findings highlight the profound influence of surface effects on thermoelastic damping. Notably, with surface effects taken into account, thermoelastic damping undergoes notable variations with the increase in film thickness for both Material I and Material II. In contrast, employing the classical continuum theory and classical thermoelectricity theory without considering the surface effect yields a consistent thermoelastic damping value.

The emergence of surface effects in thin films under periodic vibrations has been extensively documented and clarified by numerous studies^20,37,38^. At nanometric scales, the prosperities of film materials are profoundly influenced by the exposed surface^37^. As structures shrink in size, the percentage of surface atoms in the composition surges, leading to a marked increase in specific surface area and specific surface energy^38^. As the film thickness approaches submicron dimensions, this elevated surface area presents a more pronounced influence on the material’s elastic attributes. High-frequency vibrations induce temperature fluctuations, with compression-induced temperature rises in sections of the film, while tensioned sections witness temperature reductions. This temperature disparity creates an uneven stress field during vibrations, manifesting as thermoelastic damping (as depicted in Fig. 2), which becomes a primary avenue for energy loss.

**Figure 6:**
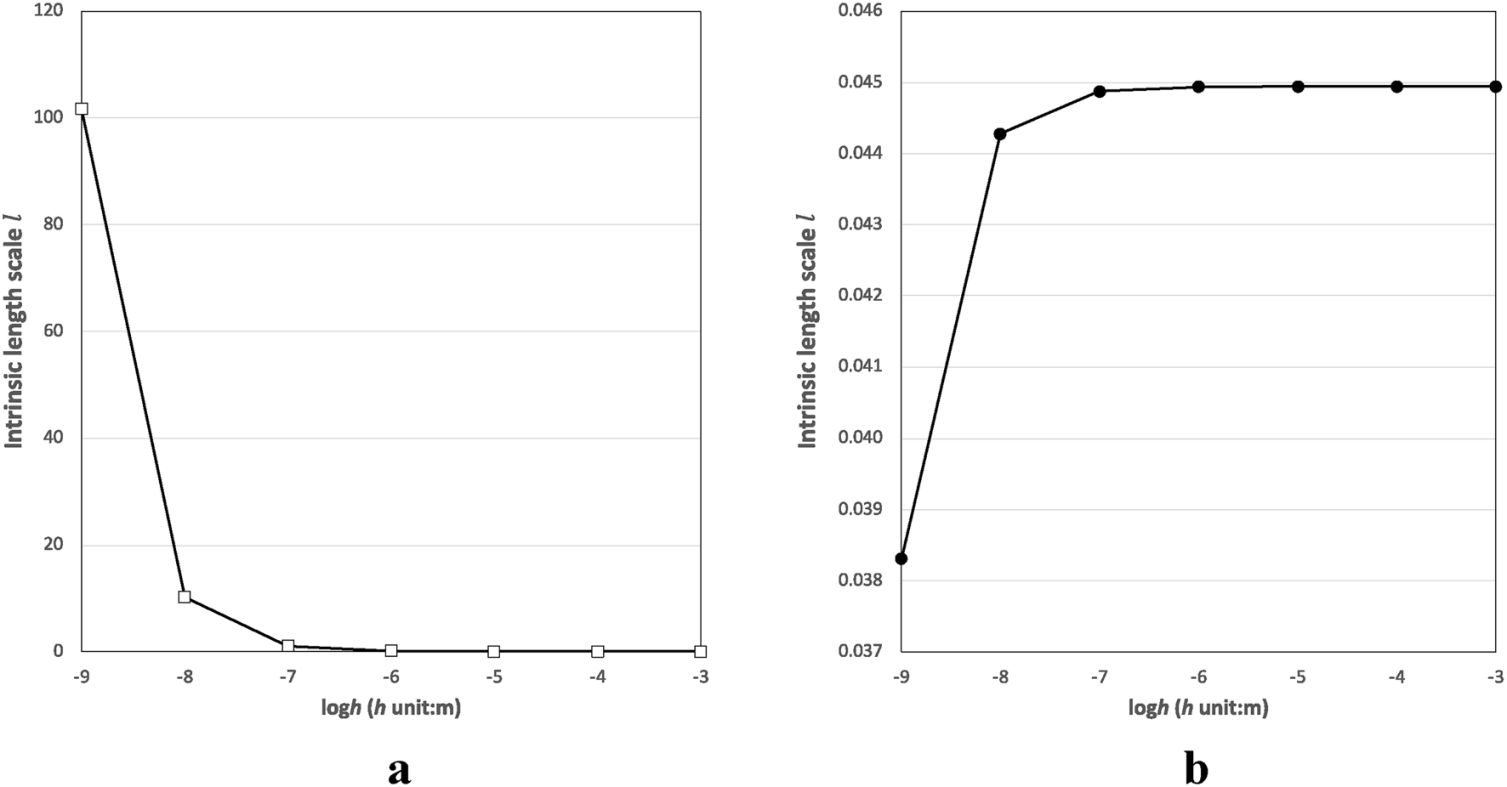
The variations of intrinsic length scale with film thickness of (**a**) Material I and (**b**) Material II.

Contrasting the thermoelastic damping variations (as illustrated in Figs. 2a and 3a) between Material I and II indicates that Material II's thermoelastic damping demonstrates exhibits less sensitivity to thickness changes under similar frequency conditions and a length-to-thickness ratio of 10 (6.54% vs. 0.06%). This diminished sensitivity in Material II can likely be linked to its considerably smaller intrinsic length scale *l* and summation terms (the key component of the denominator term in Eq. 30 for calculating thermoelastic damping. In the thermoelastic damping formula (Eq. 30), the intrinsic length scale $l = \left( {2\mu_{0} - \tau_{0} + \lambda_{0} } \right)/E$ pertains solely to the material's surface parameters. Given that the material constants of Material II are markedly smaller than those of Material I, as the thickness escalates from 10^−9^ to 10^−3^ m, the summation term for both materials, specifically $\frac{1}{{24\left( {1 - v^{2} } \right)}} + \frac{1}{4}\frac{l}{h}$, undergoes variations. For Material I, this figure drops sharply from 101.778 to 0.045, whereas for Material II, there's a minor increase from 0.038 to 0.045 (as seen in Fig. 6). Such outcomes reveal that, when surface material properties are predominant, Material II showcases reduced sensitivity to thickness alterations concerning its thermoelastic damping behavior.

However, for thinner films (namely, s = 20), the behavior alters: Material II demonstrates increased sensitivity to thickness variations in comparison to Material I, with values of 13.04% against 1.63%. This finding illustrates that the behavior of thermoelastic damping in relation to film thickness might depend on multiple elements, including the material composition and the range of thickness variation. This is supported by the fact that the material properties and width-to-thickness ratio play critical roles in the denominator and numerator of Eq. (30), respectively, which is utilized for calculating thermoelastic damping^39,40^.

The strikingly divergent trends in thermoelastic damping observed between Materials I and II can be partially attributed to their distinct surface constants. Material II possesses considerably smaller material constants in contrast to Material I. Notably, Material II's λ_0_ even presents a negative value. This leads to contrasting variation patterns in the intrinsic length scale concerning the film thickness for Materials I and II, as depicted in Fig. 6.

Our previous research^20^ revealed that the dimensionless intrinsic frequency of forcibly vibrated ultra-thin films (covering both Material I and Material II) with simple support undergoes an increase with the reduction of film thickness due to surface effect. The present study extends this understanding, illustrating the role of the forced vibration frequency in influencing the thermoelastic damping trends relative to film thickness (as presented in Figs. 4 and 5). As previously highlighted, multiple factors, including material property and the range of thickness variation, influence a thin film's thermoelastic damping behavior concerning its thickness. Therefore, it's logical that the trends in thermoelastic damping variation with film thickness under different forced vibration frequencies display contrasting patterns: the shifts in thermoelastic damping relative to film thickness due to the surface effect are more pronounced at elevated forced vibration frequencies for Material II, but they are less for Material I.

Currently, there's growing interest in investigating nanofilms and nanoplates as potential oscillators in MEMS or NEMS systems^41,42^. However, when pondering the potential application of the formula developed in this paper, it's essential to recognize that the model presumes a homogenous, isotropic, and perfectly elastic body. Future inquiries can delve into thin films that exhibit non-uniformities in both thickness and composition.

## Conclusions

In this study, we introduce a refined model to theoretically determine the thermoelastic damping of ultrathin elastic films with nanoscale thickness, considering the impact of the surface effect. Our approach hinges on numerical experiments using two distinct materials. The advanced model integrates surface stresses derived from elastic surface theory, rooted in the foundation of Kirchhoff’s kinetic hypothesis. Thermoelastic damping resulting from both surface effects and periodic vibration is determined by examining thermal dissipation alongside the maximum elastic potential energy.

Our derived explicit solution emphasizes the profound impact of surface effects on the film's thermoelastic damping. A comparative analysis between Material I and Material II unveils that Material II's thermoelastic damping exhibits reduced sensitivity to thickness alterations under similar conditions, namely frequency and a length-to-thickness ratio of 10. This is potentially due to Material II's smaller intrinsic length scale and summation terms. Nonetheless, thin film's thermoelastic damping behavior, in relation to its thickness, is also influenced by other factors, like the inherent material properties, the range of thickness variation, and the externally imposed vibration frequency.

The findings from this research offer valuable perspectives on the thermoelastic damping characteristics of thin films across various conditions. They hold significant relevance for advancing nanoscale oscillators in MEMS or NEMS systems. Future research directions could encompass the exploration of thin films characterized by irregularities in thickness and material composition.

## Data Availability

All data, models, and code generated or used in this study are available upon request from the corresponding author.
